# Maize *Gametophytic factor* loci *Ga3* through *Ga11* modify reproductive barriers

**DOI:** 10.1093/genetics/iyag242

**Published:** 2026-09-10

**Authors:** Elli P Cryan, Brandon S Gaut

**Affiliations:** Department of Ecology and Evolutionary Biology, University of California, Irvine, Irvine, CA 92697, United States; Department of Systems Biology, University of California, Irvine, Irvine, CA 92697, United States; Department of Ecology and Evolutionary Biology, University of California, Irvine, Irvine, CA 92697, United States; Department of Systems Biology, University of California, Irvine, Irvine, CA 92697, United States

**Keywords:** maize genetics, gametophytic factor, modifier, reproductive barrier, epistasis, plantae, non-Mendelian inheritance

## Abstract

Gametophytic factor (Ga) barriers are maize (*Zea mays* ssp. *mays*) reproductive barriers controlled by molecular incompatibilities between pollen and silks. Twelve distinct *Ga* loci have been identified in maize populations since the first genetic evidence of a Ga barrier was reported in 1901. Of the twelve, however, only 3 have been validated by modern molecular, functional, and genomic studies: *Ga1*, *Ga2*, and *Tcb1*. The remaining “higher” *Ga* loci, spanning *Ga3* to *Ga11*, were reported in the historical literature, but their associated phenotypes segregated in unexpected ways or disappeared over subsequent generations. Here we introduce and explore the hypothesis that the higher Ga loci represent modifiers of Ga1, Ga2, and Tcb1 barrier functions. By revisiting the historical literature, we found that barrier phenotypes fall into 2 phenotypic and functional categories. Phenotypically, the 2 categories represent healthy pollen with a silk-length effect and unhealthy pollen without a silk-length effect. These phenotypic categories were supported by genomic data; we identified candidate genes in each higher *Ga* locus by comparing historical linkage mapping data to the corresponding genomic sequence of maize reference line B73. We discovered candidate genes related to 2 broad pathways: pollen tube growth and RNA-directed DNA methylation. We conclude that the past century of evidence aligns with our hypothesis that maize loci *Ga3* through *Ga11* modify rather than directly control Ga barriers. This brief investigation provides a starting point for geneticists and evolutionary biologists to explore how strong reproductive barriers are shaped by epistatic interactions.

## Introduction

Reproductive barriers reduce or prevent gene flow among individuals, populations, and species. These barriers are central to the process of speciation (Dobzhansky 1937; Ledyard Stebbins 1950) but pose practical challenges for plant breeding by limiting the movement of desirable alleles across genetic backgrounds (e.g. Goodman et al. 2021; Moreels et al. 2023). Painstaking research has led to the identification of barriers across many plant species (Baack et al. 2015; Christie et al. 2022), including maize (*Zea mays* ssp. *mays*), which is uniquely situated as both a genomics and a genetics model system (Coe 2001; Hufford et al. 2021) with an unusually long history of research on barrier loci (Nelson 1994).

Early maize geneticists recognized that some controlled crosses failed, revealing genetically controlled incompatibilities that act after pollination but before fertilization (Correns 1902). Among the best characterized of these is the Gametophytic factor (Ga) reproductive barrier system, which has been studied for over a century (Correns 1901; Correns 1924; Jones 1924; Mangelsdorf and Jones 1926). Each *Ga* locus controls a molecular incompatibility between maize pollen and silks (maize pistils). Female plants with an active *Ga* allele express silk enzymes that disrupt the growth of haploid pollen tubes lacking a matching *Ga* allele (Moran Lauter et al. 2017). As a result, pollen tubes carrying *ga* alleles often fail to reach the ovules, preventing fertilization of *Ga* female gametes at the base of the silk (Lu et al. 2014). Plants with active *Ga* alleles may completely exclude incompatible *ga* pollen, constructing a strong reproductive barrier. This barrier is unidirectional, however, because *Ga* pollen can grow down *ga/ga* silks. *Zea* species have unusually long silks that may have contributed to the evolution and persistence of these pollen-pistil barriers, and the age of *Ga* loci suggests the barriers may have played a role in *Zea* genus diversification (Cryan et al. 2025).

Around 12 *Ga* loci have been mapped to maize chromosomes, but only 3 of those loci have been identified at the molecular level in *Zea mays* genome assemblies. The 3 validated loci are *Ga1*, *Ga2*, and *Teosinte Crossing Barrier1* (*Tcb1*) (Burnham 1936; Brieger 1937; Schwartz 1950; Evans and Kermicle 2001). Each of these encodes a matching pair of pectin methylesterase (PME) genes, one expressed in the silks and the other in pollen tubes. The ability of these PME genes to create and overcome a reproductive barrier has been validated *in planta* (Moran Lauter et al. 2017; Zhang et al. 2018, 2023; Lu et al. 2019; Chen et al. 2022) and has prompted a search for related PME-like sequences in genome assemblies at the remaining *Ga* loci (Bapat et al. 2023; Cryan et al. 2025). Unfortunately, this search has proven unsuccessful. Although there is now a firm understanding of *Ga1*, *Ga2*, and *Tcb1* haplotype diversity across modern maize lines and wild relatives, sequence-based searches have not revealed any new PME-encoding *Ga* loci in *Zea* genomes (Cryan et al. 2025).

The absence of additional PME-like loci represents a genetic mystery: what are the “higher” *Ga* loci? *Ga3* through *Ga11* were identified over decades by renowned maize geneticists including Emerson, Rhoades, Brieger, and Nelson (Brieger et al. 1938; Emerson 1944; Brieger 1945; Rhoades 1948; Schwartz 1951; Jiménez T. and Nelson 1965; Gonella and Peterson 1975; Fowler 2003). They and others used linked markers to map the “higher” *Ga* loci to genetic linkage groups (see Supplementary File 1 for citations and details). However, follow-up studies have often concluded that the loci were not present in future generations resulting from additional crosses (e.g. Ashman 1981; Nelson 1994). These mysterious disappearances, and the lack of genomic evidence of more than 3 PME-encoding *Ga* loci, contrast starkly with the quality and number of reports identifying new *Ga* loci.

What might explain the inability to find the 9 or so higher *Ga* loci? We can think of 3 possibilities. The first is that the PME sequences have not yet been found because there are not enough genome assemblies available. Both *Ga1* and *Tcb1* display some amount of presence/absence variation across over 30 genome assemblies of maize individuals and other *Zea* species (Cryan et al. 2025). If the higher-numbered *Ga* genes are present at low population frequencies, then they may be absent in the assemblies assayed thus far. Moreover, many of the geneticists who identified higher *Ga* loci worked with maize germplasm from across Central and South America, capturing a broader pool of genetic diversity than the maize genomes assembled to date (e.g. Brieger 1945; Clark 1954; House 1957; Jiménez T. and Nelson 1965). However, at least some of the higher *Ga* loci come from the core of modern maize germplasm. For example, *Ga7* was identified in crosses between B73 and a high-oil Chinese inbred line (Pan et al. 2012). In crosses between B73 and the set of 25 NAM parental inbreds, all of which have assembled genomes, there was significant segregation distortion around chromosomal regions corresponding to most of the higher *Ga* loci (McMullen et al. 2009) [See Fig. 3 in McMullen et al. 2009 for distortion regions and Table 1 for higher *Ga* locations]. Hence, one explanation for why *Ga3-Ga11* sequences remain elusive—insufficient samples of assembled genomes—seems unlikely to completely account for the missing *Ga* loci.

**Table 1.** Summary of the higher Ga loci phenotypes and converted mapping regions of Ga3 to Ga10, along with our best candidate gene and pathway for each locus.

Locus	*Ga3*	*Ga4*	*Ga5* (Brieger *Ga4*)	*Ga6*	Ga7	Ga8	*Ga9*	*Ga10*	*Ga11*
Chromo-some arm	9S	1S	4L	1S	3L	9S	4S	5S	9S
Silk-length effect	Yes (Brieger *et al*. 1938)	No (Wright 1949)	Yes (Brieger 1945)	none reported	N/A (Rhoades 1948) No (Fowler 2003)	Yes^a^ (Fowler 2003) No^a^ (Bianchi 1957)	none reported	No (Fowler 2003)	Yes (Fowler 2003)
Pollen grain defects	none reported	Yes (Emerson 1946)	No (Brieger 1945)	none reported	No (Rhoades 1943)	none reported	none reported	none reported	none reported
Mapping region size (cM)	∼2 cM in 1938; ∼1.6 cM now	∼1 cM in 1949; ∼1.3 cM now	<1 cM in 1945; <1 cM now	∼2 cM in 1963; ∼3 cM now	∼1 cM in 1966; ∼2.2 cM now	∼2 cM in 1960s; ∼2.3 cM now	∼2 cM in 1965; ∼2.7 cM now	∼1 cM in 1975; <1 cM now	N/A
B73 v5 genomic region corresponding to mapping region	chr9: 15,498,400 .. 16,093,910	chr1: 41,473,024 .. 42,367,257	chr4: 190,929,149.. 190,981,561	N/A	chr3: 236,184,856 .. 236,860,701	chr9: 20,432,209 .. 20,704,533	chr4: 180,569,234 .. 181,911,442	chr5: 17,005,166 .. 17,604,192	N/A
Number of B73 v5 gene models in mapping region	22	20	3	N/A	27	9	30	14	N/A
Best candidate modifier gene(s)^1^	VPP6 (V-PPase) or HPAT3^2^	ALY1	V-ATPase subunit	N/A	Expansin	HXXXD-type Acyltransferase	DLR2 aka TONSOKU protein	RNA Pol II subunit RPB2	N/A
Function of candidate modifier gene	vacuolar proton pump regulating pH or post-translational modifications essential for pollen tube growth	export mRNAs, including AGO and RNA Pol-encoding mRNAs, from the nucleus	vacuolar proton pump regulating pH, especially in elongating cells	N/A	loosen silk cell walls	modify plant metabolites, including cell wall components	maintain repressive epigenetic marks during DNA replication and repair	synthesize RNA, including as part of 24nt siRNA production	N/A
Tissue-specific expression of candidate modifier gene	For both: No, but highest expression in elongating cells like pollen and roots	Yes: Zygote, early embryo, sperm cell	No, but highest in silk and female spikelet	N/A	Yes: Anther, mature pollen	Yes: Silk	No, but highest in ear primordium and apical cell of the bicellular zygote	Yes: Zygote, female gametophyte, sperm cell	N/A
Candidate pathway	Cell wall growth	24nt-siRNA RdDM	Cell wall growth	N/A	Cell wall growth	Cell wall growth	24nt-siRNA RdDM	24nt-siRNA RdDM	N/A

Ga6 is excluded from most rows because it may be synonymous with Ga4; Ga11 is excluded from most rows because an exact mapping region has not been reported.

^a^See Supplementary File 1 for details about each locus. Footnote 1: candidate genes are those with expression in the appropriate tissue, see Supplementary File 1 for reasoning. Footnote 2: Two genes had expression in the expected tissue, so we report 2 “best” candidate genes for Ga3.

A second possibility is that the higher *Ga* loci do not exist—i.e. they were mistakenly detected as genomic loci that affect Ga barrier phenotypes. This possibility also appears unlikely, given that maize geneticists have consistently reported Ga barrier activity beyond the 3 validated loci. In fact, some geneticists, including Rhodes, Emerson, and Brieger, expanded their discoveries by constructing crosses between plants with and without barrier function and mapped new *Ga* loci to specific chromosomal arms using linked markers, often down to the centiMorgan (Table 1) (see Supplementary File 1 for a full list of citations). The authority, volume, and specificity of these mapping experiments suggest that most or all of the higher *Ga* loci do affect Ga barrier phenotypes, but how?

Here we investigate a third hypothesis, which is that the higher *Ga* loci are modifiers of the Ga barriers encoded by *Ga1*, *Ga2*, or *Tcb1*. We base this hypothesis on the reasoning presented in Fig. 1; the idea, briefly, is that populations homozygous for a *Ga* allele but with variation in a modifier locus will segregate for the Ga barrier phenotype. The barrier phenotype will map to the modifier locus. We propose, then, that barriers created by the *Ga1, Ga2*, or *Tcb1* loci can be mapped to unlinked modifier loci. This suggests that *Ga3-Ga11* loci encode barrier modifier genes, rather than directly encoding barrier PMEs. Given this hypothesis, we address 3 questions. Does the wealth of reports on *Ga* loci provide evidence that the barrier phenotype is modifiable by unlinked genetic loci? If so, what kind of functions might modifiers perform? Finally, by investigating the reference B73 genomic sequence at the mapping regions for *Ga3*-*Ga11*, can we identify candidate modifier genes consistent with those functions? After reviewing all Maize Newsletter reports dating from 1929 to 2020, and numerous publications about the maize *Ga3* through *Ga11* loci, we propose that higher *Ga* loci are present and encode factors that modify the strength of Ga barriers, specifically genes that affect pollen growth and the epigenetic state of barrier loci.

## Results and discussion

### Ga barrier strength can be modified

Substantial evidence indicates that the strength of the *Ga1*, *Ga2*, and *Tcb1* barriers can be modified by unlinked loci. Variation in *Ga1* barrier strength has been consistently attributed at least in part to genetic factors (Nelson 1952; Ashman 1975; González et al. 2012; Shrestha 2016; Boerman et al. 2021), and several candidate *Ga1* modifier loci have been identified through GWAS (Hurst 2019). In a population of more than 3 hundred popcorn lines, Hurst (2019) detected significant associations between Ga1 barrier strength and loci on chromosomes 2, 3, 4, 5, and 10 (see Supplementary File 1 for lack of overlap between these and *Ga* loci). Ga2 barrier strength is similarly subject to “…one or more modifying factors,” including a higher *Ga* factor, *Ga8* (Longley 1961; see Supplementary File 1 tabs for details about each locus, including *Ga8*). Tcb1 barrier strength is also modified by genetic background. While active *Tcb1* alleles can be easily introduced into a *Ga1/Ga1* background, the same is not true in the standard *ga1/ga1* background of inbred line W22 (Lu et al. 2014). When an active *Tcb1* allele was introgressed into W22, the barrier weakened progressively over 10 generations of backcrossing and was eventually abolished (Lu et al. 2014). This loss was reversible. When a mutation in *Mop1* was introduced into W22 lines lacking the *Tcb1* barrier, the barrier phenotype was restored in a subset of *mop1/mop1* and *Mop1/mop1* plants (Lu et al. 2019). And unlike active *Ga1* alleles, the *ga1* allele in the standard W22 inbred background seems to produce pollen 24-nt siRNAs that could play a role in RNA-directed DNA methylation (RdDM) of *Ga1* and *Tcb1* alleles (Cryan et al. 2025). *Mop1* encodes an RNA-dependent RNA polymerase that functions in some RdDM pathways (Alleman et al. 2006). Additional Tcb1 modifier loci have been mapped across diverse maize lines to chromosomes 1, 3, 4, 5, 7, 9, and 10 through GWAS and QTL analyses (Khaipho-Burch 2018; Maharjan 2021; see Supplementary File 1). Together, these studies have established that unlinked loci readily modify the strength of the *Ga1*, *Ga2*, and *Tcb1-*encoded barriers.

### Two potential modifier functions: pollen tube growth and RNA-directed DNA methylation

Given that the Ga1, Ga2, and Tcb1 barriers can be modified, how do modifier genes change the barrier phenotype? The reported phenotypes of the higher *Ga* loci fall into 2 categories, which may hint at modifier functions. The first phenotypic category includes Ga barriers that are overcome by healthy-looking pollen, but that exhibit a silk-length effect, meaning that longer silks are correlated with a stronger barrier. The second phenotypic category includes Ga barriers overcome by phenotypically aberrant pollen whose performance shows no silk-length effect (Table 1). In symmetry with the 2 Ga3-Ga11 phenotypic categories, 2 possible modifier functions stand out (Fig. 2). Because the Ga barrier mechanism relies on PME enzymes that disrupt pollen tube growth down the silk, one obvious candidate function is controlling pollen tube growth epistatically with PMEs. A diploid paternal plant carrying a pollen tube growth modifier could produce pollen grains that appear healthy and exhibit defects only after germination. In principle, such modifiers could be identified through GWAS and QTL studies in populations with strong Ga barriers that are weakened by a modifier allele that impairs pollen tube growth. Nearly all prior research on Ga barrier modifiers has focused on this type of population—i.e. strong barrier populations. Based on QTL evidence, several authors have proposed that unlinked genes affecting pollen tube growth can modify barrier strength (Khaipho-Burch 2018; Hurst 2019; Maharjan 2021). If modifiers alter pollen tube growth, which relies on highly expressed pollen-specific genes (Zeng et al. 2024), we expect the wild-type alleles of these pollen tube growth loci to be highly expressed in developing or mature pollen.

The change in *Tcb1* barrier strength observed in *mop1* backgrounds points to a second category of modifiers: genes that function in the RNA-directed DNA Methylation (RdDM) pathway. Modifiers of this type could influence barrier strength by altering DNA methylation of *Ga* alleles, thereby affecting both their expression and their function. Several lines of evidence are consistent with this idea. Ga1 barrier strength can vary from fully active to completely abolished, even among maize lines encoding identical *Ga1* PME genes (Cryan et al. 2025). In this case, barrier strength correlates with mRNA expression level, DNA methylation status, and the abundance of 24-nt siRNAs homologous to *Ga1* pistil PMEs (Cryan et al. 2025). *Tcb1* barrier strength also correlates with mRNA expression and DNA methylation status (Lu et al. 2019). Notably, modifiers that block RdDM and thereby restore *Ga* locus activity—such as *Mop1*—can impair maize pollen development, producing plants with visibly defective pollen grains (e.g. Teng et al. 2020).

Thus far, the only RdDM gene confirmed to modify Ga barrier repression is *Mop1*, based on the *Tcb1* study of Lu et al. (2019). Had Lu et al. mapped the *Tcb1* barrier trait in their population segregating for Tcb1 barrier reactivation, it possibly would have mapped to the *Mop1* locus (Fig. 1), though future research would be required to confirm this. Identifying additional RdDM pathway genes that modify Ga barriers will ideally require GWAS and QTL studies in populations carrying *Ga* alleles that are strongly repressed and therefore produce no Ga barrier. Basically, geneticists interested in modifications that reactivate a repressed barrier will start with a population expressing no barrier and screen for individual plants with a reactivated barrier. To our knowledge, no modifier search has deliberately focused on such a population, largely because the epigenetic repression of Ga barriers has only recently become apparent (Cryan et al. 2025). However, among the modern maize lines surveyed, more carry a repressed *Ga1* allele than an active one (Cryan et al. 2025). As a result, although to our knowledge no geneticists have deliberately screened for barrier reactivation, many have likely grown mapping populations with repressed barrier loci and genetic variation at modifier loci.

**Fig. 1.**
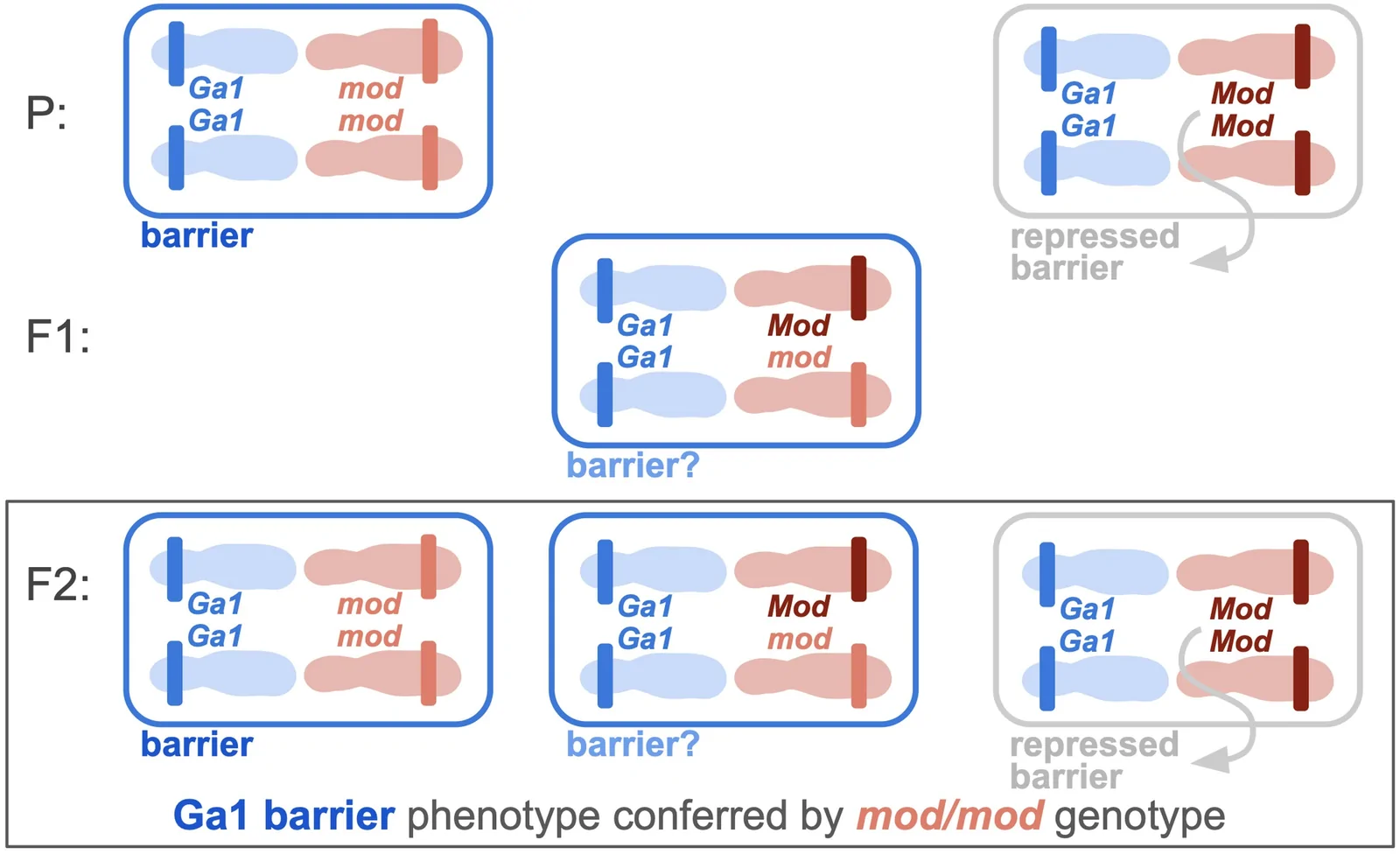
A segregating Ga barrier phenotype may map to a locus that modifies Ga1 barrier activity. In populations homozygous for *Ga1* alleles, variation may still exist at other loci. For example, in the parental (P) generation, both parents are *Ga1/Ga1* but they have opposing homozygous genotypes at a theoretical modifier locus *Mod*. In this example, plants with a *Ga1/Ga1 mod/mod* genotype express the Ga1 barrier, but the active *Mod* allele decreases barrier strength, abolishing the barrier (“repressed barrier”) in homozygous *Mod/Mod* plants. An (F1) hybrid of these 2 parent genotypes will be homozygous for the *Ga1* allele. Whether this plant encodes a barrier (i.e. “barrier?”) depends on the dominance effects of *Mod* in silks or its haploid effect in pollen. Dominance depends on the specific modifier, but generally loss of function *mod* alleles might be recessive in diploid silk but have a strong effect in haploid pollen (see Fig. 2). Selfing the F1 creates a new (F2) generation with uniform *Ga1/Ga1* genotypes but varying *mod/mod*, *Mod/mod*, and *Mod/Mod* genotypes. Because *Ga1/Ga1 Mod/Mod* plants have no barrier, the Ga1 barrier phenotype will segregate. This segregating barrier phenotype will be mapped not to *Ga1* but instead to the *Mod* locus. In any generation, the barrier phenotype can be determined by controlled crosses with *ga/ga* lines carrying a dominant kernel marker.

### Identifying candidate modifier genes from genome assemblies

We have argued that the *Ga1, Ga2,* and *Tcb1*-encoded barriers can all be modified by unlinked loci [Fig. 1], and that these modifiers may act through either pollen tube growth or 24-nt siRNA-directed DNA methylation (Fig. 2). This reasoning leads to an explicit hypothesis: the higher *Ga* loci, *Ga3* through *Ga11*, should encode genes related to these 2 functions. Is there evidence to support this hypothesis? If so, what are the candidate modifier genes?

**Fig. 2.**
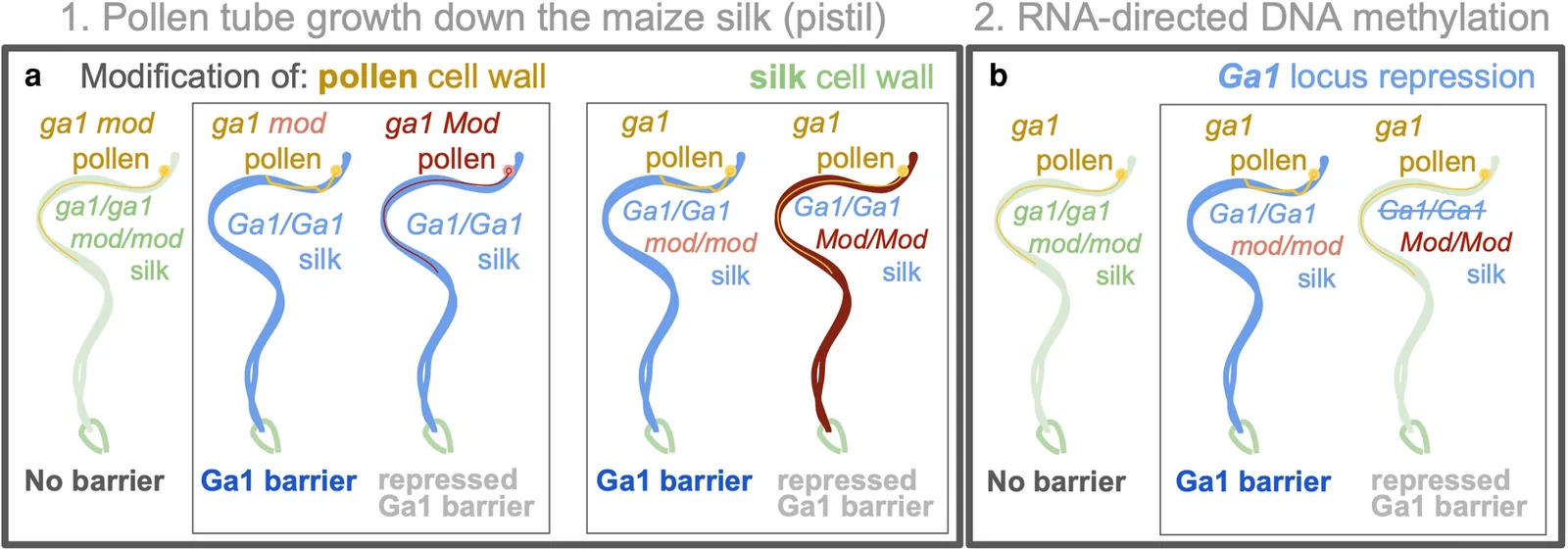
Hypothetical Ga barrier modifier functions include 1. pollen tube growth down the silk and 2. RNA-directed DNA methylation of *Ga* loci. On wildtype *ga1/ga1* silks, *ga1* pollen grows down the silk towards the egg. Silks are colored by Ga1 and Modifier expression: blue indicates Ga1 barrier PME expression, green indicates no expression, and dark red indicates both Ga1 barrier PME and Modifier expression in the silk tissue. Pollen grains are colored by Modifier expression, with yellow indicating no expression and red indicating Modifier expression in the pollen. a) Modifiers impacting pollen tube growth down the silk may impact the pollen or the silk cell well. In the case of pollen cell wall modifiers, the performance of *ga1* pollen on *Ga1/Ga1* silks is determined by a pollen allele: *mod* or *Mod*. Silk cell wall modifiers may also change the success of pollen tubes growing down *Ga1/Ga1* silks, with *mod/mod* conferring a normal Ga1 barrier and *Mod/Mod* counteracting *Ga1* to confer a weak or eliminated barrier (“repressed Ga1 barrier”). b) RNA-directed DNA Methylation (RdDM) impacts Ga1 barrier function, and genes involved in 24-nt siRNA RdDM pathways may help repress *Ga1* alleles. *Ga1/Ga1* plants express a silk barrier in *mod/mod*, but in *Mod/Mod* backgrounds, *Ga1* is repressed.

To identify candidate genes, we first determined where each higher *Ga* locus falls on the modern maize genetic map. Primary reports describe the conferred phenotype and chromosomal location of *Ga3*, *Ga4*, *Ga5*, *Ga7*, *Ga8*, *Ga9*, *Ga10*, and *Ga11* loci; chromosomal location but not phenotype data is also available for *Ga6* (see Supplementary File 1 for an annotated bibliography by locus). Some of these reports appeared in peer-reviewed journals, but many were documented only in the Maize Newsletter. *Ga4*, for example, was first reported in the 17th volume (1943) (Emerson 1943), and *Ga11* was named in the 77th (2003) (Fowler 2003). Across this 60-year span, geneticists measured the distance between *Ga* loci and nearby markers in centiMorgans (Table 1). Almost all the markers used in those historical studies are still in use, and precise genomic coordinates are now available for them. Their positions on the maize genetic map have shifted gradually, however, as map resolution has improved. As a result, while we assume each *Ga* locus retains its position relative to nearby markers, the absolute centiMorgan distance from the start of its chromosome has changed. We updated the reported map positions of the higher *Ga* loci by scaling each one by the same factor required to convert the historical position of its nearby markers to their current position on the consensus genetic map (see Supplementary File 1 for calculations) (Coe 2008; Woodhouse et al. 2025).

The updated mapping region sizes (in cM) of *Ga3* to *Ga10*, along with their locations, are presented in Table 1. The sizes of the regions range from <1 to 3 cM, and the corresponding B73 version 5 genome assembly contained from 3 to 30 gene models (Table 1). To check if the annotated gene functions align with our modifier hypothesis, we performed a cursory survey of these gene models, recognizing the limitation that the B73 genome could lack causative loci. For each *Ga* locus, we picked a “best” candidate gene by checking public B73 transcriptomic data for candidate gene expression in relevant reproductive tissues (Table 1, Supplementary File 1) (Woodhouse et al. 2025, 2021). For example, *Ga7* is reported to impact pollen grain fitness on silks with and without Ga barrier activity, so we selected the candidate gene with strong expression in male reproductive tissues (anther and mature pollen) and no expression in silks (Table 1) (see *Ga* locus tabs in Supplementary File 1 for expression profiles of all genes in the mapping regions). *Ga3* had 2 candidate genes with expression in male reproductive tissues, where we expect Ga3 phenotype activity, so we report 2 “best” candidates (Table 1). Based on this criterion, we found potential modifier genes at each *Ga* locus region consistent with 1 of the 2 expected functions: (1) pollen tube growth down the silk or (2) 24-nt siRNA-directed DNA methylation pathways.

Interestingly, these 2 candidate gene functions also match the 2 barrier phenotype categories. The higher *Ga* loci that contain pollen tube growth pathway candidate genes fall into phenotype category 1, with healthy-looking pollen and a silk-length effect. Plants with these barriers produce apparently normal pollen grains and show a stronger barrier when the silks are longer. Because the Ga barrier mechanism is the failure of *ga* pollen tubes to grow to the embryo sac at the bottom of a *Ga* silk, longer silks can give *ga* pollen an even lower chance of overcoming a Ga barrier. Pollen tube growth rate down the silk can be impacted by cell wall phenotypes of the growing pollen tube or the silk itself (Fig. 2), and genes highly expressed in maize pollen vegetative cells often contribute to pollen-pistil interactions (Warman et al. 2020). Higher *Ga* loci in phenotype category 1 include *Ga5* and *Ga8*. There are only 3 gene models in the 1cM *Ga5* mapping region (Table 1). The best candidate of those 3 encodes a vacuolar ATPase subunit most highly expressed (>200 FPKM) in female spikelets and silks (Table 1) (Woodhouse et al. 2025, 2021). Vacuolar ATPases play an important role in transport of cell wall materials during directed cell growth (Seidel 2022). Similarly, the best candidate gene in the *Ga8* mapping region encodes a silk-expressed acyltransferase that could in theory epistatically interact with Ga barrier PMEs. Longley (1961) noted that rather than encoding an independent Ga barrier, *Ga8* could instead be “modifying the eliminating character” of *Ga2*, the only PME-encoding Ga locus for which RdDM has not been reported (Longley 1961). (Nelson later reported in response that unlike *Ga8*, *Ga2* itself does not require an unlinked modifying locus to create a barrier. This supports our idea that *Ga2* directly encodes a barrier while *Ga8* does not (Nelson 1962).)

By contrast, the higher *Ga* loci related to RdDM pathways fall into phenotype category 2 (unhealthy pollen and no silk-length effect). Since some types of RdDM can play an important role in the development of plant reproductive tissues (Chow and Mosher 2023), certain plants deficient in some RdDM and demethylation pathway proteins have low fitness gametophytes, resulting in visibly wrinkled and unfilled pollen (Pal et al. 2024, Zeng et al. 2024). The lack of silk-length effect also matches our proposal that some higher *Ga* loci modify epigenetic repression of a PME-encoding Ga barrier. Silk length affects the chance that *ga* pollen will reach the bottom of a *Ga/Ga* or *Ga/ga* silk. Silk-length effect is difficult to observe when the Ga barrier is very strong, the *ga* pollen is in competition with *Ga* pollen, or both. In populations where Ga barrier function maps to a segregating modifier allele, we expect the genotype at the *Ga* locus to be homozygous *Ga/Ga* (Fig. 1). Epigenetic modification has been observed for Ga1 and Tcb1 (Lu et al. 2014, 2019; Cryan et al. 2025), both of which are very strong barriers when unrepressed (Schwartz 1950; Kermicle 2006). When a Ga1 or Tcb1 barrier is at full strength, *ga* pollen exclusion can be up to 100%, and silk-length effects are almost impossible to observe. If epigenetic repression leads to the lack of silk-length effect in the second phenotypic category, then the 2 broad categories of higher Ga barrier phenotypes match our 2 hypothesized categories of modifiers. A potentially compelling example of category 2 is the Ga4 barrier, which displays defective pollen and no silk-length effect (Table 1) (Emerson 1946; Wright 1949). The *Ga4* mapping region includes the candidate gene ALY1 (also known as Tho complex subunit 4), which exports mRNAs essential for RdDM in arabidopsis (Choudury et al. 2019) and interacts with chromatin at an allele that plays a role in homology-dependent silencing via 24-nt siRNAs in maize (Lynn et al. 2025)

## Conclusions

While these results are encouraging, the purpose of this Brief Communication was to investigate, using largely historical observations, a long-standing genetic phenomenon. Clearly, more focused studies on each locus are necessary to confirm that the higher *Ga* loci really do modify Ga1, Ga2, and Tcb1 barriers. Moreover, our reliance on historical data is likely to limit resolution; although some of the map regions are very narrow and contain only 3 B73 gene models, some higher *Ga* loci could not be assigned a single best candidate and any “real” candidate could be lacking from the B73 assembly. Additionally, we have not validated the map locations of the higher *Ga* loci with specific crosses, and some will prove impossible because the appropriate lines were never saved, have been lost, or have been dropped from the Maize Genetics Cooperation Stock Center catalog (e.g, Ga6 stock was lost by 1980 (Coe 1980) while Ga4 stock was lost and Ga9 stock was lapsed by 1984 (Hoisington and Coe 1984)). As a result, we cannot be sure that our candidate genes control the phenotypes observed in the original Maize Newsletter reports. One potential route forward is genetic modification. Although CRISPR is possible in maize (Svitashev et al. 2016), testing all of these candidate genes in controlled backgrounds and with the appropriate alleles at *Ga1*, *Ga2* and *Tcb1* loci is likely to take many years. Nonetheless, understanding the genetic control of the Ga barriers is important, because it may help breeders better control pollen contamination in maize with active barriers, like most popcorns, many lines developed with tropical germplasm, and many traditional varieties from Central and South America (Nelson 1952; Goodman et al. 2021). And while reproductive barriers are present across the plant tree of life (Baack et al. 2015, Christie et al. 2022), their genetic basis can be difficult to disentangle, especially in ecological or evolutionary model systems without the same genomic and genetic resources as maize. From what we do understand about the unusually well-studied barriers of maize, they may have played a role in enabling the diversification of the *Zea* genus (Cryan et al. 2025) and may still play a role in maintaining the subspecies identities of maize and its wild relatives (Berube et al. 2024; Cryan et al. 2025), but these conclusions are recent and somewhat preliminary. Ultimately, understanding the genetic basis of reproductive barriers will help evolutionary geneticists better understand whether barriers like Ga regularly contribute to reproductive isolation and ultimately speciation.

## Methods

### Literature search

We identified as many reports on maize *Ga* loci or barriers as possible by searching through the maize literature, beginning with papers and reports cited by classic *Ga1* and *Ga2* papers. Many observations of *Ga* loci and maize populations displaying Ga barrier phenotypes were reported in the Maize Newsletter (aka MNL, now hosted on MaizeGDB) (Woodhouse et al. 2025). MNL reports were often brief summaries of crossing results, so to avoid missing any descriptions of Ga phenotypes or Ga segregation in reports that preceded the naming of a locus, we read all reports on segregation distortion of any trait. We made note of any reports with descriptions of segregation distortion and linked markers or crossing results indicating a Ga reproductive barrier was at play. We then narrowed our search only to Ga barriers not mapped to the *Ga1*, *Ga2*, or *Tcb1* mapping regions. We were unable to find primary reports on *Ga6*, but found multiple sources reporting mapping locations, phenotypes, or segregation patterns of the remaining “higher” Ga barriers (Ga3-Ga11). Papers and MNL reports relevant to *Ga3-Ga11*, as well as unnamed *Ga* loci, are listed in Supplementary File 1.

### Identifying two categories of barrier phenotypes

After reading all sources listed in Supplementary File 1, we compiled reported phenotypes of barriers Ga3-Ga11. Geneticists working on Ga barriers reported phenotypes including plant appearance, segregation of linked markers, barrier strength across generations, pollen and silk appearance, pollen viability, controlled crosses and phenotypes and counts of the resulting seed. Of these phenotypes, two are especially important to the classification of a segregation distortion locus as a Ga barrier: (i) the viability of pollen and (ii) the segregation of linked markers in seeds across the ear as a result of silks at the base of the ear being longer. Phenotypes seemed distinct, with some barriers matching the healthy pollen and silk-length effect phenotype also typical of Ga1, Ga2, and Tcb1 barriers, while other barriers that showed no silk-length effect were not reported as having unhealthy pollen (Table 1, Supplementary File 1).

### Identifying *Ga3-Ga11* genomic regions

Crossing data and map locations have been reported for all loci *Ga3-Ga11* (Supplementary File 1). To compare modern genomic data to mapping data, we converted the reported map locations to coordinates in the B73 v5 reference genome using the 2008 Genetic map maintained and updated by MaizeGDB (Coe 2008; Woodhouse et al. 2025). Specifically, for each locus, we recorded the reported cM distance between linked markers flanking the *Ga* locus. Almost all linked markers used in the literature are also used today, and both the high-resolution map location and the genomic coordinates of the underlying gene model are included in the Genetic map. We converted each Ga locus from the historical to the modern mapping region by scaling the distance from the linked marker loci by the change in the historical to the modern map locations of the linked loci (see tabs for each *Ga* locus in Supplementary File 1 for all calculations and explanations). To avoid over-interpreting the map locations, we used the significant figures of the original linkage data to maintain the same level of uncertainty in our scaled modern map locations. If map distances expanded, as is often the case, our uncertainty in the *Ga* locus mapping region also expanded. Mapping regions reported for *Ga6* and *Ga11* were respectively not consistent and not narrow enough for us to confidently report an updated mapping region (see Ga6 and Ga11 tabs of Supplementary File 1 for details).

### Identifying *Ga3-Ga11* candidate genes

After obtaining updated B73 v5 genomic coordinates for higher *Ga* loci, we downloaded all gene models in each region from MaizeGDB (Woodhouse et al. 2025). We counted the number of gene models (Table 1) and then checked expression patterns of each gene model using the MaizeGDB expression visualization tool qTeller (Woodhouse et al. 2021). Supplementary File 1 provides qTeller links for every gene model. Based on the phenotypic descriptions of each locus, we picked predicted tissues, such as pollen or silk tissues, where we expected underlying genes to be expressed (Supplementary File 1). Genes expressed in those tissues were considered “best” candidate genes, with only 1 or 2 genes in each window matching expected expression patterns (Table 1, Supplementary File 1). We next checked functional annotations for each candidate gene (Table 1) by performing a cursory literature search (Supplementary File 1). Candidate genes fell into 2 broad pathways, reported in Table 1.

## Supplementary Material

iyag242_Supplementary_Data

## Data Availability

The authors affirm that all data necessary for confirming the conclusions of this article are represented fully within the article, its tables and figures, and its supplemental material. Expression data visualizations were accessed using the qTeller tool from MaizeGDB and can be accessed through links in Supplementary File 1 or viewed as static files on GSA FigShare (https://doi.org/10.25386/genetics.32680908). Supplemental material available at GENETICS online.
