# Supplementary material for: Maize Gametophytic factor loci Ga3 through Ga11 modify reproductive barriers
Source: Genetics. 2026 Sep 10;234(3):iyag242. doi: 10.1093/genetics/iyag242 (PMC13732701; doi:10.1093/genetics/iyag242)
Supplement: Supplementary file 1 [file iyag242_supplementary_data.zip › Supplemental_References_GENETICS-2026-309887.docx]

**Supplemental Material: Literature Cited**

[Ashman, R. B. 1981. “Failure to Verify the Ga9 Locus on Chromosome 4.” *Maize Genetics Cooperation Newsletter* 55.](http://paperpile.com/b/2bNTbh/zwPqm)

[Bauman, L. F. 1954. “Results of Further Inheritance Studies of the Kys Type of Cytoplasmic Male Sterility in Corn.” *Maize Genetics Cooperation Newsletter* 28.](http://paperpile.com/b/2bNTbh/Tlyea)

[Beckett, J. B., C. R. Burnham, E. H. Coe, M. P. Maguire, E. B. Patterson, and R. L. Phillips. 1978. “Cytogenetic Working Map.” *Maize Genetics Cooperation Newsletter* 52.](http://paperpile.com/b/2bNTbh/Om1Jo)

[Bianchi, Angelo. 1957. “Ga Factors in Maize-Teosinte Derivatives.” *Maize Genetics Cooperation Newsletter* 31.](http://paperpile.com/b/2bNTbh/D7gdC)

[Bianchi, Angelo. 1958. “Ga Factors in Maize-Teosinte Derivatives.” *Maize Genetics Cooperation Newsletter* 32.](http://paperpile.com/b/2bNTbh/TA2tG)

[Bianchi, Angelo. 1972. “Gametophyte Factors in Chromosome 9 of Inbred Lines.” *Maize Genetics Cooperation Newsletter* 46.](http://paperpile.com/b/2bNTbh/kwQqM)

[Bianchi, Angelo, and M. R. Parlavecchio. 1965. “A Possible Study of Crossing-over on the Basis of Pollen Traits.” *Maize Genetics Cooperation Newsletter* 39.](http://paperpile.com/b/2bNTbh/UI0Pu)

[Bianchi, Angelo, and M. R. Parlavecchio. 1966. “Location of Ga8 in Chromosome 9 Linkage Group.” *Maize Genetics Cooperation Newsletter* 40.](http://paperpile.com/b/2bNTbh/wWonG)

[Bianchi, Angelo, and M. R. Parlavecchio. 1967. “Relationship between Gametophyte Factors and Markers of Chromosome 9.” *Maize Genetics Cooperation Newsletter* 41.](http://paperpile.com/b/2bNTbh/wrPmC)

[Bianchi, Angelo, and M. R. Parlavecchio. 1968. “Further Data on Location of a Ga Factor on Chromosome 9.” *Maize Genetics Cooperation Newsletter* 42.](http://paperpile.com/b/2bNTbh/hhCFq)

[Bianchi, Angelo, and M. R. Parlavecchio. 1969. “Further Data on the Ga Factor of Chromosome 9.” *Maize Genetics Cooperation Newsletter* 43.](http://paperpile.com/b/2bNTbh/MQVyo)

[Brieger, F. G. 1945. “Estudos Genéticos Sôbre O Milho Tunicata.” *Anais Da Escola Superior de Agricultura Luiz de Queiroz* 2 (0): 209–238.](http://paperpile.com/b/2bNTbh/xlZWO)

[Brieger, F. G. 1947. “Collection of Indigeneous Corn.” *Maize Genetics Cooperation Newsletter* 21.](http://paperpile.com/b/2bNTbh/bQSmb)

[Brieger, F. G., G. E. Tidbury, and H. P. Tseng. 1938. “Genetic Control of Gametophyte Development in Maize: II. The Quarter Test.” *Journal of Genetics* 36 (1): 17–38.](http://paperpile.com/b/2bNTbh/JMm4D)

Brüx, Angela, Tzu-Yin Liu, Melanie Krebs, York-Dieter Stierhof, Jan U. Lohmann, Otto Miersch, Claus Wasternack, and Karin Schumacher. 2008. “Reduced V-ATPase Activity in the Trans-Golgi Network Causes Oxylipin-Dependent Hypocotyl Growth Inhibition in Arabidopsis.” *The Plant Cell* 20 (4): 1088–1100.

[Burnham, C. R. 1952. “Linkage Tests.” *Maize Genetics Cooperation Newsletter* 26.](http://paperpile.com/b/2bNTbh/0GTBr)

[Clark, E. 1954. “Gametophyte Factors in Pop Corns.” *Maize Genetics Cooperative Newsletter* 28.](http://paperpile.com/b/2bNTbh/fXWJ3)

[Coe, E. H. 1980. “A Revision of the Working Linkage Map for Chromosome 3.” *Maize Genetics Cooperation Newsletter* 54.](http://paperpile.com/b/2bNTbh/KemIq)

[Coe, E. H., Jr. 1980. “Zealand 1980.” *Maize Genetics Cooperation Newsletter* 54.](http://paperpile.com/b/2bNTbh/pd4Mu)

Cosgrove, Daniel J. 2015. “Plant Expansins: Diversity and Interactions with Plant Cell Walls.” *Current Opinion in Plant Biology* 25 (June): 162–72.

[Emerson, R. A. 1943. “Aberrant Pericarp-Color Ratios.” *Maize Genetics Cooperation Newsletter* 17.](http://paperpile.com/b/2bNTbh/AviXq)

[Emerson, R. A. 1944. “Aberrant Pericarp-Color Ratios.” *Maize Genetics Cooperation Newsletter* 18.](http://paperpile.com/b/2bNTbh/5u7kj)

[Emerson, R. A. 1946. “Ga_4_ and Pericarp-Color Ratios.” *Maize Genetics Cooperative Newsletter* 20.](http://paperpile.com/b/2bNTbh/aaL77)

[Ford, Leola E. 1949. “2.” *Maize Genetics Cooperation Newsletter* 23.](http://paperpile.com/b/2bNTbh/yInWJ)

[Fowler, J. E. 2003. “A Gametophyte Factor on Chromosome 9 Affects Both Male and Female Gametophytes.” *Maize Genetics Cooperation Newsletter* 77.](http://paperpile.com/b/2bNTbh/1f2kB)

Gao, Yonggang, and Cheng Zhao. 2024. “Utilizing Multi-Omics to Elucidate the Characteristics of Male Sterility in the Anthers of the Maize Ms712c Sterile Line.” https://doi.org/10.2139/ssrn.5031897.

[Gonella, J., and P. A. Peterson. 1975. “Gametophyte Factor (ga10) on Chromosome 5 Distal to A2.” *Maize Genetics Cooperation Newsletter* 49.](http://paperpile.com/b/2bNTbh/6btnc)

[Hannah, L. Curtis. 1976. “Characterization of a Gametophyte Factor-like System on Chromosome 5.” *Maize Genetics Cooperation Newsletter* 50.](http://paperpile.com/b/2bNTbh/wDgJQ)

[Hoisington, D. A., and E. H. Coe Jr. 1984. “Mapping: Gene List.” *Maize Genetics Cooperation Newsletter* 58.](http://paperpile.com/b/2bNTbh/gt0Jj)

[House, L. R. 1957. “Gametophyte Factors in Maize.” *Maize Genetics Cooperative Newsletter* 31.](http://paperpile.com/b/2bNTbh/xVwVl)

[Jimenez T., Jorge, and O. E. Nelson. 1962. “A Test for Fifth Chromosome Gametophyte Factors in Some Mexican Races.” *Maize Genetics Cooperation Newsletter* 36.](http://paperpile.com/b/2bNTbh/OW9wi)

[Jiménez T., Jorge R., and Oliver E. Nelson. 1965. “A New Fourth Chromosome Gametophyte Locus in Maize.” *The Journal of Heredity* 56 (6): 259–263.](http://paperpile.com/b/2bNTbh/AUCZj)

[Knott, D. R. 1963. “Chromosome 1 Linkage Data.” *Maize Genetics Cooperation Newsletter* 37.](http://paperpile.com/b/2bNTbh/MgNVi)

Li, Daojun, Yudong Wang, Yan Li, Wenli Wang, Chunfei Wang, Xiaoqing Wang, Wenqi Zhou, et al. 2025. “Maize ZmDLR2/BRU1 Is Required for Lateral Root Primordium Emergence by Participating in DNA Repair.” *Plant, Cell & Environment* 48 (10): 7377–91.

[Longley, A. E. 1961. “A Gametophyte Factor on Chromosome 5 of Corn.” *Genetics* 46 (6): 641–647.](http://paperpile.com/b/2bNTbh/7Q8Pj)

Luo, Bowen, Ziqi Zhang, Binyang Li, Haiying Zhang, Junchi Ma, Jing Li, Zheng Han, et al. 2024. “Chromatin Remodeling Analysis Reveals the RdDM Pathway Responds to Low-Phosphorus Stress in Maize.” *The Plant Journal: For Cell and Molecular Biology* 117 (1): 33–52.

Lynn, Jason S., Kathryn M. Koirtyohann, Yacob B. Gebreab, Jaliyah Edwards, and Karen M. McGinnis. 2025. “Identification of Proteins Associated with Stably Integrated Maize B1 Tandem Repeat Transgene Chromatin.” *Plants*14 (12): 1863.

MacAlister, Cora A., Carlos Ortiz-Ramírez, Jörg D. Becker, José A. Feijó, and Zachary B. Lippman. 2016. “Hydroxyproline O-Arabinosyltransferase Mutants Oppositely Alter Tip Growth in Arabidopsis Thaliana and Physcomitrella Patens.” *The Plant Journal: For Cell and Molecular Biology* 85 (2): 193–208.

[Miranda, Luiz Eugenio Coelho de, and Luiz Torres de Miranda. 1992. “Allometric Genetics X. A Three Point Test Involving a Gametophyte Gene (*Ga10 A2 Bt*).” *Maize Genetics Cooperation Newsletter* 66.](http://paperpile.com/b/2bNTbh/IJsD9)

Molina, Isabel, and Dylan Kosma. 2015. “Role of HXXXD-Motif/BAHD Acyltransferases in the Biosynthesis of Extracellular Lipids.” *Plant Cell Reports* 34 (4): 587–601.

[Mouli, Chandra, and N. K. Notani. 1968. “A New Gametophyte Factor on Chomosome 9.” *Maize Genetics Cooperation Newsletter* 42.](http://paperpile.com/b/2bNTbh/qLRVJ)

[Nelson, O. E. 1962. “A Test for an Unlinked Inhibitor of a 5th Chromosome Gametophyte Factor.” *Maize Genetics Cooperative Newsletter* 36.](http://paperpile.com/b/2bNTbh/NRjBu)

[Nelson, Oliver E. 1960. “The Wx/wx Locus.” *Maize Genetics Cooperation Newsletter* 34.](http://paperpile.com/b/2bNTbh/YZXzf)

[Nelson, Oliver E. 1994. “The Gametophyte Factors of Maize.” In *The Maize Handbook*. Springer New York.](http://paperpile.com/b/2bNTbh/ZHWOt)

[Neuffer, M. G. 1966. “Linkage Maps of Maize Chromosomes.” *Maize Genetics Cooperation Newsletter* 40.](http://paperpile.com/b/2bNTbh/uhLBr)

Onodera, Yasuyuki, Kosuke Nakagawa, Jeremy R. Haag, Diane Pikaard, Tetsuo Mikami, Thomas Ream, Yusuke Ito, and Craig S. Pikaard. 2008. “Sex-Biased Lethality or Transmission of Defective Transcription Machinery in Arabidopsis.” *Genetics* 180 (1): 207–18.[Rhoades, M. M. 1943. “Gametophyte Factor in Chromosome 3.” *Maize Genetics Cooperation Newsletter* 17.](http://paperpile.com/b/2bNTbh/I46B5)

[Rhoades, M. M. 1948. “Gametophyte Factor in Chromosome 3.” *Maize Genetics Cooperation Newsletter* 22.](http://paperpile.com/b/2bNTbh/7gwVN)

[Schwartz, D. 1951. “Gamete Factor.” *Maize Genetics Cooperation Newsletter* 25.](http://paperpile.com/b/2bNTbh/LitMp)

[Schwartz, D. 1952. “Gamete Factor on Chromosome 9.” *Maize Genetics Cooperation Newsletter* 26.](http://paperpile.com/b/2bNTbh/h4P5O)

[Schwartz, Drew. 1950. “Male Sterility.” *Maize Genetics Cooperation Newsletter* 24.](http://paperpile.com/b/2bNTbh/3Zwdw)

[Sprague, G. F. 1994. “Stocks and New Factors.” *Maize Genetics Cooperation Newsletter* 68.](http://paperpile.com/b/2bNTbh/vAl6i)

[Stinard, Philip. 2015. “Off Ratio Chromosome 6 Trait Recovered from Maiz Colorado Cuarenton.” *Maize Genetics Cooperation Newsletter* 89.](http://paperpile.com/b/2bNTbh/Ap0VL)

Stroud, Hume, Maxim V. C. Greenberg, Suhua Feng, Yana V. Bernatavichute, and Steven E. Jacobsen. 2013. “Comprehensive Analysis of Silencing Mutants Reveals Complex Regulation of the Arabidopsis Methylome.” *Cell* 152 (1–2): 352–64.

[Ting, Y. C. 1961. “Cross-Sterility in Chalco Teosinte.” *Maize Genetics Cooperation Newsletter* 35.](http://paperpile.com/b/2bNTbh/fSZbB)

Valenzuela-Apodaca, Juan P., Abraham Cruz-Mendívil, Grethel P. Gaytán-Pinzón, Hervey Rodríguez-González, Luis A. Peinado-Fuentes, Eduardo Sandoval-Castro, and Carlos L. Calderón-Vázquez. 2022. “Identification of SNP Markers Associated to Protein and Oil Content in Doubled Haploid Maize Lines Derived from Subtropical Breeding Populations.” *Tropical Plant Biology* 15 (4): 326–38.

Wang, Lin, Mande Xue, Huairen Zhang, Lijun Ma, and Danhua Jiang. 2023. “TONSOKU Is Required for the Maintenance of Repressive Chromatin Modifications in Arabidopsis.” *Cell Reports* 42 (7): 112738.

Wang, Yaqiong, and Hong Ma. 2015. “Step-Wise and Lineage-Specific Diversification of Plant RNA Polymerase Genes and Origin of the Largest Plant-Specific Subunits.” *The New Phytologist* 207 (4): 1198–1212.

[Wright, J. E., Jr. 1949. “Ga4 and Pericarp‑color Ratios.” *Maize Genetics Cooperation Newsletter* 23.](http://paperpile.com/b/2bNTbh/LAtbH)

Xie, Kailing, Guoqing Wang, Ying Ni, Minghui Shi, Lixue Sun, Beijiu Cheng, and Xiaoyu Li. 2025. “ZmHPAT2 Regulates Maize Growth and Development and Mycorrhizal Symbiosis.” *Plants* 14 (10): 1438.
